# One Health to Advance Tuberculosis Elimination in Brazil: A New Area of the Brazilian Tuberculosis Research Network

**DOI:** 10.1590/0037-8682-0063-2026

**Published:** 2026-09-18

**Authors:** Fernando Nogueira Souza, Jane Megid, Marcos Bryan Heinemann, Franciarli Silva da Paz, Erica Chimara, Ricardo Alexandre Arcêncio, José Roberto Lapa e Silva

**Affiliations:** 1 Universidade de São Paulo, Faculdade de Medicina, Instituto de Medicina Tropical, São Paulo, SP, Brasil.; 2 Universidade Estadual Paulista, Faculdade de Medicina Veterinária e Zootecnia, Botucatu, SP, Brasil.; 3 Universidade de São Paulo, Faculdade de Medicina Veterinária e Zootecnia, São Paulo, SP, Brasil.; 4 Rede Brasileira de Pesquisas em Tuberculose, REDE-TB, Rio de Janeiro, RJ, Brasil.; 5 Instituto Adolfo Lutz, São Paulo, SP, Brasil.; 6 Universidade de São Paulo, Escola de Enfermagem de Ribeirão Preto, Ribeirão Preto, SP, Brasil.; 7 Universidade Federal do Rio de Janeiro, Faculdade de Medicina, Rio de Janeiro, RJ, Brasil.

Zoonotic tuberculosis (zTB), caused by the *Mycobacterium tuberculosis* complex (MTC), exemplifies interconnected human-animal-environmental health threats. Globally prioritized as a human disease, zTB remains marginalized when it involves animals or the environment, despite its bidirectional transmission and likely underestimation^1^.

Estimates suggest that zTB causes tens of thousands of human cases each year. The World Health Organization estimates that more than 140,000 new cases and more than 12,000 deaths are attributable to*M. bovis* globally annually. However, the reported numbers almost certainly underestimate the true burden. Routine diagnostic workflows seldom distinguish*MTC* species in clinical practice, leading to systematic misclassification of zoonotic infections as conventional TB. Accordingly, the true incidence and outcomes of zTB remain poorly defined^2^ ^,^ ^3^.

Underestimation is particularly pronounced in regions where close contact among humans, domestic animals, wildlife, and contaminated environments coincides with persistent consumption of unpasteurized milk. Therefore, reporting zTB as a small proportion of all tuberculosis (TB) cases further underestimates its public health relevance. Even a fraction of the approximately ten million TB cases estimated annually worldwide translates into a substantial absolute burden, often borne by marginalized populations with limited access to diagnosis and care^2^ ^,^ ^3^.

Furthermore, clinical outcomes are frequently worse than those associated with*M. tuberculosis*, particularly for infections caused by*M. bovis*, which is intrinsically resistant to pyrazinamide, one of the four cornerstone drugs of standard first-line TB regimens, and is more commonly associated with extrapulmonary disease, a pattern that contributes to persistent diagnostic blindness and leads to inadequate early treatment^3^.

Beyond *M. bovis*, *M. tuberculosis* can infect livestock, wildlife, and pets (dogs and cats, including those infected with multidrug-resistant strains), enabling reverse zoonoses^1^ ^,^ ^4^ ^-^ ^6^. Wildlife reservoirs (e.g., badgers and possums), together with environmental persistence, complicate control, whereas nontuberculous mycobacteria interfere with diagnostic tests. *M. tuberculosis*infections in pets are often interpreted as isolated clinical events, without systematic epidemiological investigations of human cases within the same household. Gaps in diagnosis and surveillance prevent the recognition of companion animal infections as evidence of ongoing intrahousehold transmission and obscure their possible role in sustaining transmission cycles in urban settings^4^ ^-^ ^6^.

Brazil faces enzootic bovine TB (prevalence ranging from 0.2-9% across states; São Paulo 9%). Despite the establishment of a structured national control program, the Brazilian National Program for the Control and Eradication of Brucellosis and Tuberculosis (PNCEBT), launched by the Brazilian Ministry of Agriculture and Livestock (MAPA) in 2001, implementation has failed to translate into sustained progress because of voluntary herd accreditation^7^, thereby increasing foodborne risks associated with the consumption of unpasteurized milk (approximately one-third of the market) and artisanal cheeses. Notably, Santa Catarina and Mato Grosso have recently implemented more intensive surveillance systems aimed at eradicating bovine TB, and their outcomes warrant close monitoring as potential models for other Brazilian states^7^.

Human cases lack MTC speciation, and occupational exposures (e.g., farmers and veterinarians) are not systematically monitored. Globally, 90% of countries lack zTB data, and Brazil's siloed surveillance reflects this gap^8^. Brazil exemplifies the challenges of zTB control in middle-income countries with large agricultural sectors and high TB burdens. Importantly, complementary initiatives have recently emerged within the human health sector, including the 2023 national clinical and surveillance guidelines for zoonotic TB issued by the Brazilian Ministry of Health^9^, although integration between sectors remains limited.

Therefore, the Brazilian Tuberculosis Research Network (REDE-TB) is well positioned to establish a dedicated working group on “*Tuberculose Zoonótica no Contexto de Uma Só Saúde - Zoonotic Tuberculosis in the Context of One Health*”, creating a national platform for multicenter studies, diagnostic innovations, and knowledge translation across the human-animal interfaces. By embedding One Health principles into its research and advocacy activities, REDE-TB could generate the evidence needed to strengthen surveillance, improve public health and veterinary practice, and inform future policy aimed at reducing the burden of zTB in Brazil.

Furthermore, REDE-TB could coordinate multicenter pilot studies on molecular MTC speciation in reference laboratories, promote harmonized protocols for case definition and reporting across human and veterinary health systems, and support continuing education for clinicians, veterinarians, and surveillance professionals. Dedicated multidisciplinary working groups could further develop consensus recommendations for household and occupational contact investigations following confirmed animal or human MTC infections, alongside risk communication strategies for populations at increased risk of zTB exposure. Collectively, these activities would position REDE-TB as a national translational research platform, accelerating evidence generation and facilitating its uptake by policymakers, professional societies, and surveillance systems.

Bridging the remaining knowledge gaps in zTB will require systematic data collection, integrated surveillance, and coordinated multidisciplinary research. Priority areas include improving diagnostic capacity in both human and veterinary medicine, elucidating transmission dynamics at the human-animal interface, evaluating treatment outcomes, and defining the role of animals as reservoirs and sentinels for infection^10^. Reducing transmission will also require evidence-based interventions targeting food safety, animal health, occupational exposure, and other risk factors for zTB.

Although the One Health approach has gained global recognition as a framework for addressing health challenges at the human-animal-environment interface, its operationalization in TB control remains limited.** **In this context, REDE-TB is well positioned to bring together expertise across human, animal, and environmental health to generate robust evidence and consensus recommendations to support the Ministry of Health, MAPA, and other stakeholders in strengthening zTB surveillance and control. Such collaborative efforts would reinforce the implementation of the One Health approach in Brazil, providing a stronger scientific foundation for evidence-informed policies and integrated TB control strategies^10^.

Sustained investment in interdisciplinary research and cross-sectoral One Health collaboration will be essential to accelerate progress toward the End TB Strategy and the long-term goal of TB elimination by 2050.

## Data Availability

Research data is available in the body of the article.
